# Revealing the Wonder of Natural Photonics by Nonlinear Optics

**DOI:** 10.3390/biomimetics7040153

**Published:** 2022-10-05

**Authors:** Dimitrije Mara, Bojana Bokic, Thierry Verbiest, Sébastien R. Mouchet, Branko Kolaric

**Affiliations:** 1 Institute of General and Physical Chemistry, Studentski trg 12/V, 11158 Belgrade, Serbia; 2 Center for Photonics, Institute of Physics, University of Belgrade, Pregrevica 118, 11080 Belgrade, Serbia; 3 Molecular Imaging and Photonics, Department of Chemistry, KU Leuven, Celestijnenlaan 200D, 3001 Heverlee, Belgium; 4 Department of Physics and Astronomy, University of Exeter, Stocker Road, Exeter EX4 4QL, UK; 5 Department of Physics & Namur Institute of Structured Matter (NISM), University of Namur, Rue de Bruxelles 61, 5000 Namur, Belgium; 6 Micro- and Nanophotonic Materials Group, University of Mons, Place du Parc 20, 7000 Mons, Belgium

**Keywords:** natural photonics, photonics, linear and nonlinearspectroscopy, fluorescence, fluorescence spectroscopy, two-photon fluorescence, second-harmonic generation, third-harmonic generation

## Abstract

Nano-optics explores linear and nonlinear phenomena at the nanoscale to advance fundamental knowledge about materials and their interaction with light in the classical and quantum domains in order to develop new photonics-based technologies. In this perspective article, we review recent progress regarding the application of nonlinear optical methods to reveal the links between photonic structures and functions of natural photonic geometries. Furthermore, nonlinear optics offers a way to unveil and exploit the complexity of the natural world for developing new materials and technologies for the generation, detection, manipulation, and storage of light at the nanoscale, as well as sensing, metrology, and communication.

## 1. Introduction

It has been known for a long time that colors in nature are not designed for beauty but are of the utmost importance for communication. Structural colors belong to a special class of colors that have no chemical origin, but they arise from the interaction of light with structures, such as periodically arranged materials [1,2]. Structural colors are ubiquitous colors among insects, fish, and birds. In addition, they are a main topic of research in fields such as biophotonics and biomimetics. In this perspective article, we demonstrate the interest and impact of nonlinear optical studies of photonic structures. We highlight the benefit of nonlinear optical techniques for revealing the details of structured matter at the nanoscale and its interaction with light. We also emphasize that the control of structural colors is essential for various applications in materials science. After introducing the readers to basic concepts of nonlinear optics and natural photonics, we present different cases of natural photonic structures (sometimes combined with artificial materials) investigated by nonlinear optical techniques.

## 2. Basics of Nonlinear Optics

Nonlinear optics is a part of optics that studies light propagation in nonlinear media. In such media, the polarization *P* has a nonlinear response to the electric field *E*. Such optical behavior usually occurs at a high intensity of light, such as the one generated by a laser.

In the linear regime, when an electromagnetic wave interacts with some materials, e.g., a medium containing electric charges, a dipolar type of interaction appears between the dipoles in the medium and the incident electromagnetic fields at a frequency ω [3]. This interaction can be described by an induced polarization $P_{\mathrm{ind}}$ that is linear with the electric field *E* of the incident light and acts as the source of radiation:

(1) $$P_{\mathrm{ind}\;}=\chi^{\left(1\right)}E,$$

with $\chi^{\left(1\right)}$, the linear susceptibility that is related to the refractive index. The response can be modelled by a classical harmonic oscillator, yielding a linear complex refractive index of the medium that scales with ω [3].

**Figure 1:**
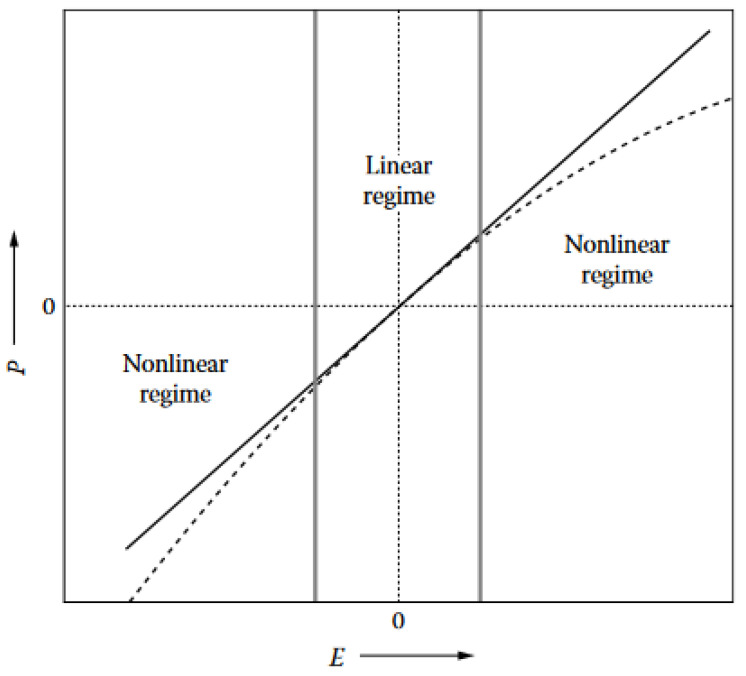
Depending on the intensity of the incident electric field (*E*), the electric polarization (*P*) will respond linearly or nonlinearly. As long as the intensity of the electric field is small, the electric polarization is linear to the electric field intensity. This case corresponds to the linear regime. When the intensity of the electric field is high, *P* is not proportional to *E* and the regime is nonlinear. Reproduced from Verbiest, T., Clays, K., and Rodriguez, V., 2009. Second-order Nonlinear Optical Characterization Techniques: An Introduction, with permission from Taylor and Francis Group, LLC, a division of Informa plc.

However, in the case of high-intensity incident electromagnetic fields, such as laser light (Figure 1), the harmonic oscillator response is not sufficient anymore to describe the observed phenomena [4,5,6,7]. The oscillations become anharmonic, i.e., they do not respond linearly to the incident electromagnetic wave. In the nonlinear regime, the induced polarization $P_{\mathrm{ind}}$ is expanded in a Taylor series as a function of the total applied electric field. The induced polarization is then written as [7]:

(2) $$P_{\mathrm{ind}}=P^{\left(1\right)}+P^{\left(2\right)}+P^{\left(3\right)}+\cdots =\chi^{\left(1\right)}E+\chi^{\left(2\right)}EE+\chi^{\left(3\right)}EEE+\cdots$$

with $P^{\left(1\right)}$, the linear part of the induced polarization; $P^{\left(2\right)}$, the second-order nonlinear response; and $P^{\left(3\right)}$, the third order nonlinear response. $\chi^{\left(1\right)}$, $\chi^{\left(2\right)}$, and $\chi^{\left(3\right)}$ are the linear, the first nonlinear, and the second nonlinear susceptibilities, respectively. The latter two quantify the second-order and third-order nonlinear optical response. Since all susceptibilities are related to the refractive index, a nonlinear complex refractive index is obtained.

One of the advantages of nonlinear optical techniques is due to the nature of the nonlinear susceptibilities, which are tensors of third and fourth rank, respectively. Especially, second-order nonlinear optical effects, which are described by the third-rank tensor $\chi^{\left(2\right)}$, have extremely interesting symmetry properties. In general, $\chi^{\left(2\right)}$ is a third-rank tensor with 27 components, but the number of independent and nonvanishing components is dependent on the symmetry of the medium. All of the components of $\chi^{\left(2\right)}$ will vanish in a medium with inversion symmetry. On the other hand, any surface where the symmetry is necessarily broken will typically yield four independent susceptibility components [7]. Since the nonlinear susceptibility directly determines the magnitude and phase of the nonlinear response, the measured response can serve as a means to evaluate the symmetry of the sample.

In addition to the optical properties of matter that can be probed by linear techniques, much more information is available by exploiting the interaction of matter with higher-intensity laser beams. Multiphoton (including two-photon) excitation fluorescence is certainly the most used nonlinear optical technique. In the case of two-photon excitation fluorescence (TPEF), high-intensity photons illuminate a sample [7]. Because of this high intensity, two photons simultaneously interact with the sample, following different selection rules from the photons in the linear light–matter interaction regime. Since two photons are absorbed initially and give rise to only one emitted photon, the resulting photon has a higher energy than each of the two absorbed photons. Multiphoton absorption and fluorescence originate from a third-order nonlinear optical response [7].

In second harmonic generation (SHG), the material also interacts with two photons [7]. Unlike two-photon excitation fluorescence, a third photon is instantaneously emitted (within ca $10^{-15}$ s) with exactly twice the energy (and, thus, half the wavelength) of the two initial photons. Since SHG is a second-order nonlinear optical process, selection rules that are different from TPEF apply: symmetry requirements such as non-centrosymmetric samples are essential to observe SHG [4,5,6,7]. Similarly, third harmonic generation (THG) corresponds to the instantaneous emission of a single photon following the interaction of three incident photons [7]. The generated photon has three times the energy and a third of the wavelength of the three initial photons.

There are multiple advantages to nonlinear techniques with respect to linear optical techniques [8,9]. An important feature is the increase in imaging depth, which can be attributed to multiple factors: first, there is a low probability of multiple photons interacting simultaneously, resulting in a minimal imaging volume at the femtoliter scale by reducing out-of-focus fluorescence. It is therefore possible to image accurately at different depths, similarly to confocal microscopes, and to create 3D reconstructions of the investigated material without the use of a pinhole. Due to the absence of a pinhole, more light can be collected and thus a clearer image can be formed. This also results in an increase in the practical resolution as the pinhole of confocal microscopes is often opened to increase the amount of incoming light to image fluorescing samples. This results in a lower resolution. Practically, the resolution of a multiphoton microscope was shown to approach 250 nm, which equals the best possible resolution of the confocal fluorescence microscopy. Another significant advantage of nonlinear optical techniques is the use of near-infrared excitation light with wavelengths corresponding to the transparency window of biological tissue. This leads to an increase in penetration depth, and it is therefore possible to image sensitive samples without damaging the structure or with minimal damage. Furthermore, most multi-photon microscopes use femtosecond-lasers as an excitation source, which further reduces the risk of photodamage. In addition, no sample preparation is necessary. This constitutes the main advantage over more complicated microscopy techniques such as scanning electron microscopy (SEM) or transmission electron microscopy (TEM) [10,11]. Finally, another interesting application of multiphoton microscopy is the imaging of the local polarization anisotropy via SHG, enabling the user to seperately determine the orientation and degree of organization of each non-centrosymmetric component within a sample.

## 3. Introduction to Natural Photonics

Colours in nature originate from chemical or physical properties of matter, light sources, or combinations of them [1,2]. Chemical colours are caused by pigments. These molecules selectively absorb incident light within a given wavelength band. Examples are eumelanins and pheomelanins, which give rise to brown/black and yellow/red human skins, respectively [12,13]. Light that is not absorbed by pigments is scattered, giving rise to colours that an observer may perceive. Physical colours are due to interference between incident light and a physical structure. Hence, they are often called structural colours, as a synonym. Structures giving rise to such colours have geometrical dimensions at the micro- or nanoscale. They encompass optical thin films [14,15,16,17], diffraction gratings [18,19,20], Bragg mirrors [21,22,23,24,25,26], chirped multilayer reflectors [27,28], and photonic crystals [29,30,31,32,33,34], as well as quasi-ordered [35,36,37,38,39,40] and randomly disordered [41,42,43,44] photonic structures.

**Figure 2:**
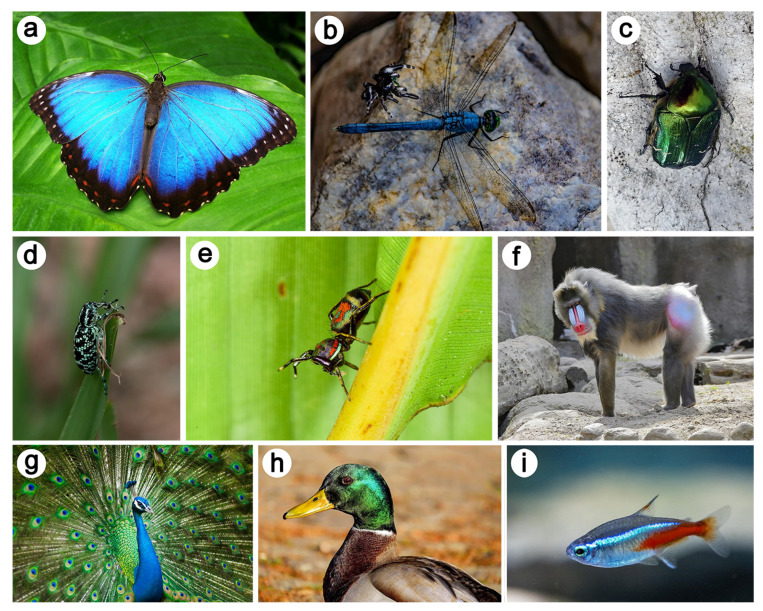
Many examples of structural colours are found in the integuments of natural organisms. They include the wings of the common morpho *Morpho peleides* (**a**), the body of some dragonfly species (**b**), the thorax and elytra of the green rose chafer *Cetonia aurata* (**c**), the body of the Botany Bay diamond weevil *Chrysolopus spectabilis* (**d**), the integuments of some jumping spider species (**e**), the skin of the mandrill *Mandrillus sphinx* (**f**), the feathers of the Indian peafowl *Pavo cristatus* (**g**), the head of the mallard *Anas platyrhynchos* (**h**), and the body of the neon tetra fish *Paracheirodon innesi* (**i**). Reproduced from from https://pixabay.com/ accessed on 27 July 2022.

Natural photonics is the field of research that studies the interaction between light and such physical structures in nature. Natural structural colours occur in organisms ranging from mammals such as primates (including human blue eyes) and marsupials, fish, or birds such as hummingbirds and pigeons to insects such as butterflies and beetles (Figure 2) [1,2,45].

This section introduces key concepts and presents some selected case studies from natural photonics, in the linear regime.

**Figure 3:**
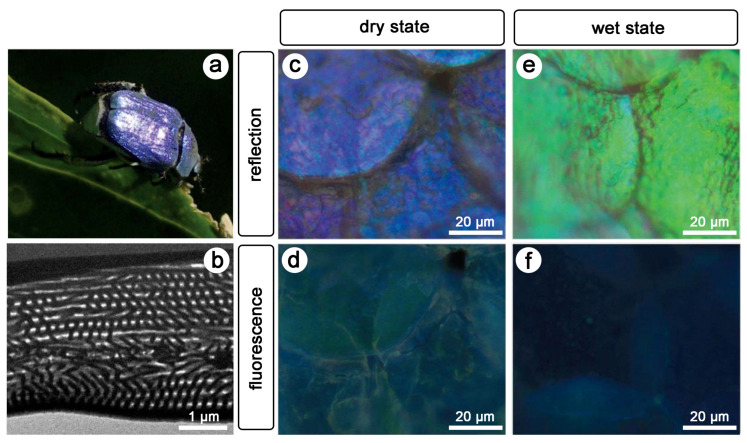
The blue-violet colour of the male beetle *Hoplia coerulea* (**a**) originates from a multilayer photonic structure. This porous periodic multilayer (**b**) occurs in the scales covering the elytra (**c**) and body of the insect. Upon UV light illumination, the scales display a turquoise colour through fluorescence (**d**). When in contact with water, the scales turn to green (**e**) and navy blue (**f**) under visible and UV lightillumination, respectively. Reproduced from Mouchet, S.R., Lobet, M., Kolaric, B., Kaczmarek, A.M., Van Deun, R., Vukusic, P., Deparis, O., and Van Hooijdonk, E., 2016. Controlled fluorescence in a beetle’s photonic structure and its sensitivity to environmentally induced changes. Proc. R. Soc. B 283, 20162334, with permission from The Royal Society.

One striking example is the case of the male beetle *Hoplia coerulea* (Figure 3). The blue-violet iridescent colour of its elytra and body observed in reflection with incident visible light arises from a photonic structure, namely, a porous periodic multilayer, within the round scales occurring on its body [46,47].

This multilayer is mainly composed of chitin, the building material of insects [1,2]. Upon contact with liquids and vapour, these scales change their colour to green [48,49,50,51,52]: the spectral reflectance peak red-shifts. This colour change arises from the penetration of some liquid into the pores of the scales, filling the pores and changing the refractive index.

Furthermore, one-photon excitation fluorescence (OPEF) emission was observed when the beetle was illuminated with UV light (Figure 3d) [53,54]. The colour of the scales covering the elytra and body of this insect is turquoise. This phenomenon arises in biological organisms, the integuments of which contain so-called fluorophores [55,56]. Examples include birds [44,57,58,59], insects [60,61,62,63,64,65,66,67,68,69,70,71], arachnids [72,73], mammals [74], amphibians [75,76], reptiles [77], marine animals [78,79], and plants [80,81] (Figure 2). These molecules emit longer-wavelength light (typically, visible photons) following the absorption of incident shorter-wavelength light (typically, UV, violet or blue photons).

**Figure 4:**
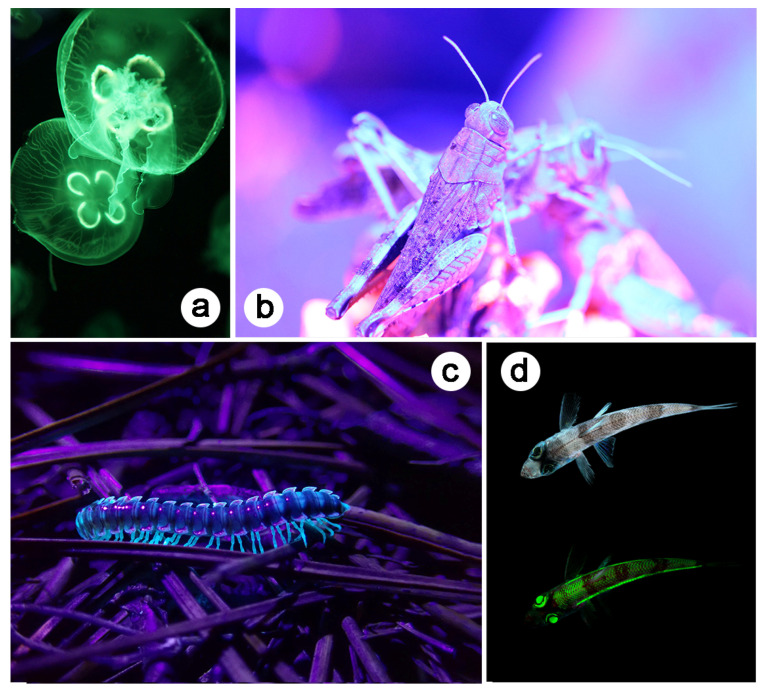
Fluorescence is ubiquitous in the integuments of natural organisms. The integuments of some jellyfish species (**a**), some grasshopper species (**b**), some millipede species (**c**), and some greeneye fish species (**d**) are known for their fluorescent properties. (**d**) Top (bottom): greeneye fish under visible (UV) light. Reproduced from from pixabay.com.

Fluorescence emission results from transition between two real electron states with the same multiplicity of spin. The role, if any, of fluorescence (Figure 4) in visual communication is unclear [55,56]. Biopterin, the green fluorescent protein (GFP), papiliochrom II, psittacofulvin, and resilin are examples of fluorophores.

When light emission takes place in photonic structures, the structure may modify the directionality, the decay time, and the spectral intensity of the emitted light [82,83]. Light emission can be reduced or even inhibited if the emission wavelength is in the range of the photonic bandgap of the structure. The decay time of the excited is then increased and can, theoretically, be infinite. When such light emitted in a photonic structure originates from fluorescence, this phenomenon is often referred to as controlled fluorescence. The confinement of fluorophores in natural photonic structures was found in several species [84,85,86,87,88], including the male beetle *H. coerulea* [53,54]. Upon contact with water, the peak in the fluorescence emission spectrum from the scales of this beetle blue-shifts, giving rise to a navy blue colour (Figure 3d,f). Following the penetration of the porous structure by water, the local density of optical states (LDOS) is modified, leading to the observed colour change.

**Figure 5:**
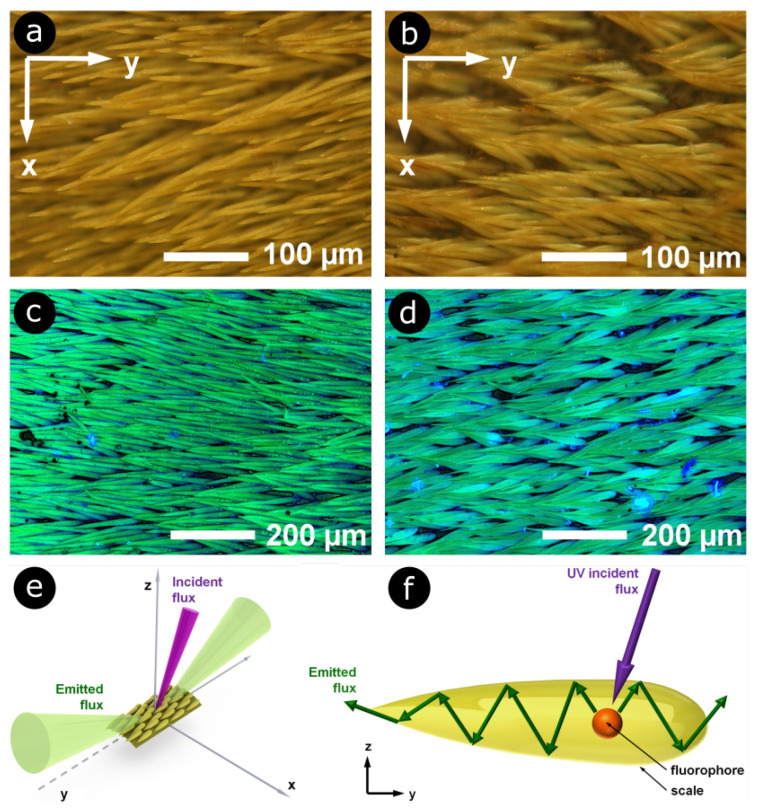
The elytra of longhorn beetles *Celosterna pollinosa sulfurea* and *Phosphorus virescens* are covered by elongated scales, in which fluorophores are embedded. These scales act as waveguides for the light emitted by the fluorophores. They display a yellow colour under visible light for *C. pollinosa sulfurea* (**a**) and *P. virescens* (**b**) (here, observation by optical microscopy). Under UV light, the observed colour is turquoise in both respective cases (**c**,**d**). Upon illumination with UV light (represented in magenta), embedded fluorophores mostly emitted within two emission cones (in green) (**e**) due to a waveguide effect taking place within the scales (**f**). Reproduced from Van Hooijdonk, E., Barthou, C., Vigneron, J.-P., and Berthier, S., 2013. Yellow structurally modified fluorescence in the longhorn beetles *Celosterna pollinosa sulfurea* and *Phosphorus virescens* (Cerambycidae). J. Lumin. 136, 313–321, with permission from Elsevier.

The interaction between fluorescent light and photonic structures was also highlighted in the scales covering the elytra of longhorn beetles *Celosterna pollinosa sulfurea* and *Phosphorus virescens* [68]. These scales exhibit yellow and turquoise colours under visible and UV incident light, respectively, with an underlying basal brown membrane (Figure 5a–d), akin to the beetles *Euchroea auripigmenta* and *Trictenotoma childreni* [69,70]. Through scatterograms and detailed series of simulations, it was demonstrated that the scales play the role of waveguides for light emitted by the embedded fluorophores (Figure 5e,f).

## 4. Nonlinear Optical Study of Natural Photonic Structures

Harnessing light–matter interaction in a nonlinear regime, researchers and engineers developed various tools for imaging and spectroscopy analyses of biological samples [8,62,89,90,91,92,93,94,95,96,97,98,99,100,101,102,103,104,105]. Nonlinear optical imaging and spectroscopy have proved to be versatile and efficient techniques in biomedical and biological research, with many benefits, including an increase in analytic depth and a reduction in photodamage, with respect to classical linear optical techniques [8]. In general, nonlinear optical studies led to a better understanding of the link between the optical response and geometries of natural photonic structures that are essential for potential applications of these structures in biomimetics and quantum technology [9].

**Figure 6:**
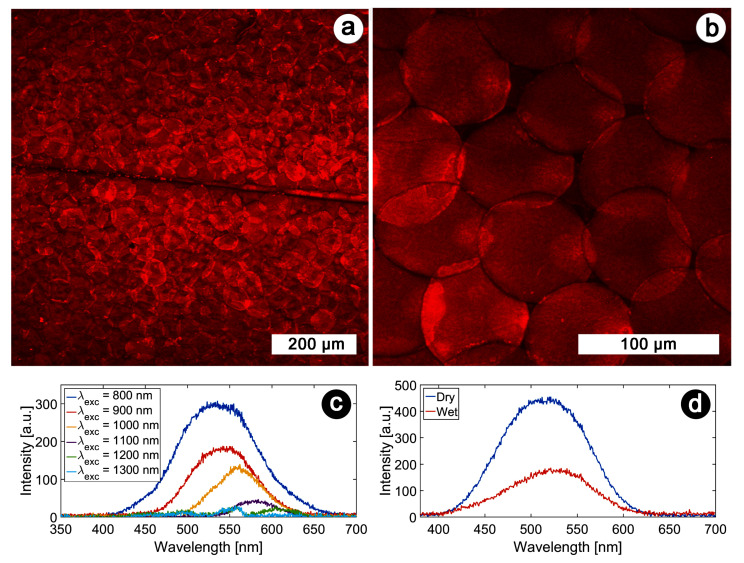
TPEF microscopy and spectroscopy of the scales covering *H. coerulea*’s elytra. An intense TPEF response was detected from the elytra of the male beetle *H. coerulea* with an excitation wavelength equal to 900 nm (**a**) and 800 nm (**b**). TPEF excitation spectra were measured with various excitation wavelengths (**c**). Upon contact with water (**d**), the emitted intensity decreases and the peak wavelength red-shifts slightly (the excitation wavelength equals 800 nm). Scale bars: (**a**) 200 µm and (**b**) 100 µm. Reproduced from Mouchet, S. R., Verstraete, C., Mara, D., Van Cleuvenbergen, S., Finlayson, E. D., Van Deun, R., Deparis, O., Verbiest, T., Maes, B., Vukusic, P., and Kolaric, B., 2019 Nonlinear optical spectroscopy and two-photon excited fluorescence spectroscopy reveal the excited states of fluorophores embedded in a beetle’s elytra, Interface Focus 9(1), 20180052, with permission from The Royal Society.

For instance, the multi-excited states character of the fluorophores embedded in the integuments of *H. coerulea* were revealed by comparing OPEF spectra with TPEF measurements [106] (Figure 6a–d).

This provided insight into the electron structure of the embedded fluorophores.

Furthermore, local-form anisotropy arising from local-direction-dependent subwavelength morphology was shown to influence both linear and nonlinear optical responses (in reflection and emission) thanks to THG spectroscopy [106]. The anisotropy is caused due to the subwavelength spacers located in the mixed air-chitin layers (Figure 3b). These spacers are locally parallel.

This finding highlighted the need to take a more accurate model into account for predicting some of the optical properties of the elytra of this beetle.

**Figure 7:**
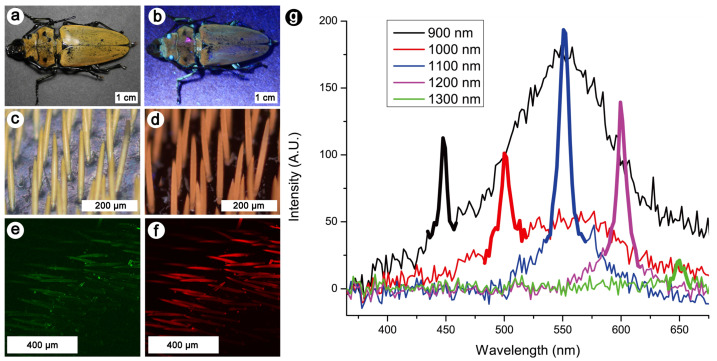
The yellow and fluorescent scales occurring on the elytra of the log-boring beetle *T. childreni* were investigated by nonlinear optical techniques, including OPEF, TPEF, and SHG microscopy and spectroscopy. The elytra exhibit a yellow colour under both incident visible white (**a**) and UV (**b**) light. This colour is due to the presence of elongated scales covering the elytra (**c**,**d**). Under visible (**c**) or UV light (**d**), they appear in shades of yellow. Upon excitation with a fundamental wavelength of 1000 nm, a SHG (**e**) and a TPEF (**f**) signal can be detected from the scales (here observed by microscopy in false colours). Multiphoton emission spectra of the log-boring beetle’s scales measured with various excitation wavelengths (**g**) exhibit SHG peaks at half the excitation wavelengths (thick lines). TPEF peaks were observed around 550 nm at most excitation wavelengths. Reproduced from Mouchet, S. R., Verstraete, C., Kaczmarek, A. M., Mara, D., van Cleuvenbergen, S., Van Deun, R., Verbiest, T., Maes, B., Vukusic, P., and Kolaric, B., 2019, Unveiling the nonlinear optical response of *Trictenotoma childreni* longhorn beetle, J. Biophot. 12(9), 12:e201800470, with permission from John Wiley and Sons.

Similarly, the yellow and fluorescent elongated scales covering the elytra of the log-boring beetle *Trictenotoma childreni* were investigated by nonlinear optical methods, including OPEF, TPEF, and SHG microscopy and spectroscopy (Figure 7) [70]. These scales appear similar to the ones of the longhorn beetles *Celosterna pollinosa sulfurea* and *Phosphorus virescens* (Figure 5) [68]. They contain fluorophores that give rise to a yellow visual appearance upon UV illumination. These observations allowed one to highlight the non-centrosymmetric nature of the fluorophores embedded within these scales [70].

**Figure 8:**
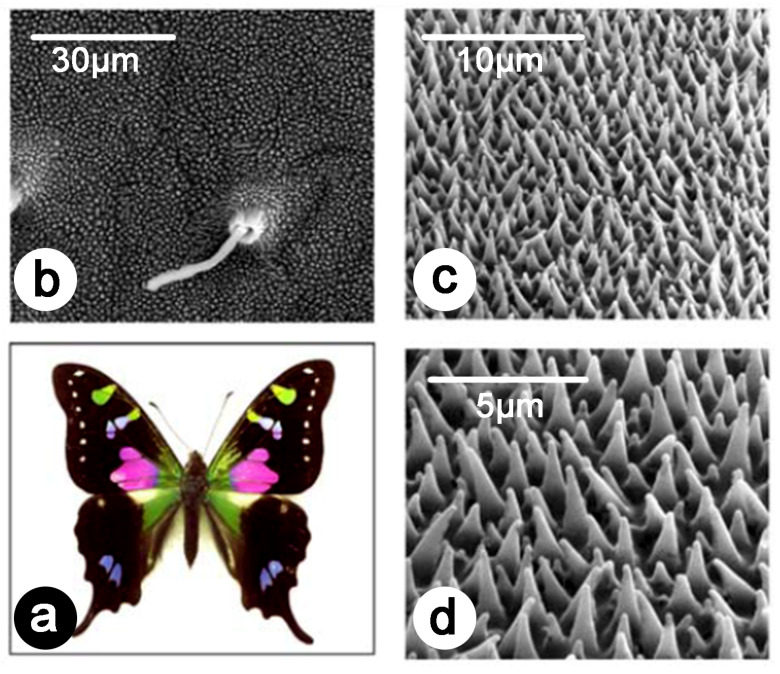
The structures occurring on the wings of the purple spotted swallowtail butterfly (*Graphium weiskei*) coated with a metal thin film were found to be an excellent substrate for SERS, in terms of biocompatibility and sensitivity [108,109,110]. *G. weiskei* (**a**) exhibit conical microstructures on its wings (**b**–**d**) as observed here by SEM. After coating by gold or silver, these structures can be used to detect protein binding from direct observation of the modifications in the SERS response. Reproduced from Garrett, N. L., Vukusic, P., Ogrin, F., Sirotkin, E., Winlove, C. P., and Moger, J., 2009, Spectroscopy on the wing: Naturally inspired SERS substrates for biochemical analysis, J. Biophot. 2(3), 157–166.

So far, various natural photonic structures from different insects, including butterflies and cicadas, have been investigated for sensing and materials-oriented applications. For example, corrugated natural photonic structures offer a unique possibility to develop new sensing platforms by combing corrugation at different scales, plasmonic properties, and surface-enhanced Raman spectroscopy (SERS) [107,108,109,110,111]. The work of Garrett and coworkers clearly shows the benefit of natural and bioinspired structuring, which can be used for different sensing applications (Figure 8) [108,109,110]. The development of such novel platforms relying on SERS will lead to the fast and accurate screening of chemical, biochemical, and pharmaceutical compounds, which is crucial for the growing fields of proteomics, genomics, molecular medicine, and biophysics, as well as for the development of assays for the detection of diseases [107,112].

The work of Stoddart and coworkers highlighted that practical applications of SERS for sensing depend on the development of manufacturing methods that will be used to mimic the complex morphology of insect integuments [107]. It pointed out the importance of biomimetics for the advancement of materials science [107]. In addition, it was shown that the enhancement of SERS also depends on the aspect ratio of metallic nanoparticles. The presence of different multiscale groove-like structures presented the possibility of developing smart surfaces for controlling optical response and wetting properties [113]. Structuring materials have a significant effect on the latter, as was demonstrated both theoretically and experimentally, specifically at different length scales [114,115,116].

**Figure 9:**
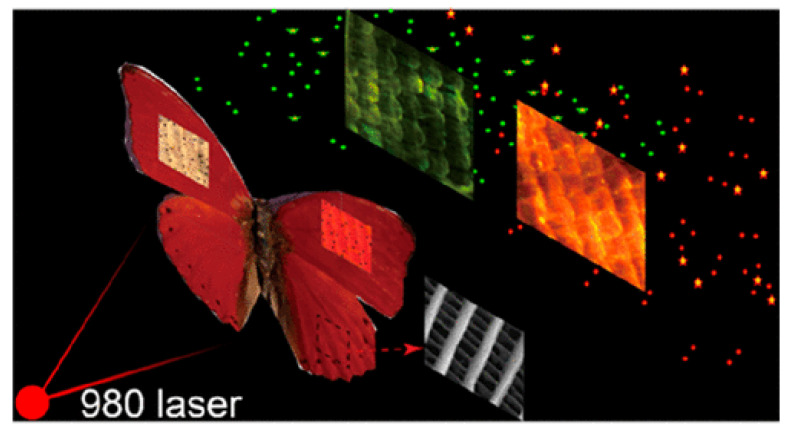
The upconversion luminescence of nanoparticles doped with lanthanide (${\mathrm{NaYF}}_{4}:{\mathrm{Yb}}^{3+},{\mathrm{Er}}^{3+}$) was controlled thanks to the natural photonic crystals occurring in the wings of the butterfly *Cymothoe sangaris*. With a 980 nm incident light, various luminescent colours were generated. Reproduced from Gao, T., Zhu, X., Wu, X. J., Zhang, B., and Liu, H. L., 2021, Selectively Manipulating Upconversion Emission Channels with Tunable Biological Photonic Crystals, J. Phys. Chem. C, 125(1), 732–739, with permission from ACS Publications.

Furthermore, the natural photonic structures occurring in the wings of the butterfly *Cymothoe sangaris* were used for tuning the upconversion luminescence of nanoparticles doped with lanthanide (${\mathrm{NaYF}}_{4}:{\mathrm{Yb}}^{3+},{\mathrm{Er}}^{3+}$) [117]. Upon illumination with a light at 980 nm, both red and green emission bands of ${\mathrm{NaYF}}_{4}:{\mathrm{Yb}}^{3+},{\mathrm{Er}}^{3+}$ could be controlled, producing different luminescent colours Figure 9. Doping natural photonic structures with materials including metals and oxides also presents the possibility of using these structures as templates to design nanocorrugated materials (with complex shapes and geometries) on demand for various material applications [118].

## 5. Conclusions

In this perspective article, we presented theoretical key concepts of nonlinear optics and discussed the results of nonlinear optical studies in the field of natural photonics. Because nonlinear optical techniques are inherently sensitive to symmetry; the presence of interfaces; and chirality, they offer a more detailed insight into the molecular properties of biomaterials. This is crucial for applications in different areas of material science. Even though, at the moment, nonlinear optical investigations of natural photonics are still at their infant stage of development, the primary aim of this article is to draw the attention of the broad material, photonic, and biological scientific communities to the capability of nonlinear optics, which could be used to forge new horizons in physical, biological and material research.
